# *catena*-Poly[(dichloridozinc)-μ-bis­(pyridin-3-yl)methanone-κ^2^ *N*:*N*′]

**DOI:** 10.1107/S160053681104671X

**Published:** 2011-11-16

**Authors:** Fan Zhang

**Affiliations:** a Department of Chemistry, Capital Normal University, Beijing 100048, People’s Republic of China

## Abstract

In the title polymer, [ZnCl_2_(C_11_H_8_N_2_O)]_*n*_, the Zn^II^ atom lies on a twofold rotation axis and has a distorted tetra­hedral ZnCl_2_N_2_ geometry involving two chloride donors and two N-atom donors from μ_2_-bridging bis­(pyridin-3-yl)methanone ligands, which also have twofold symmetry. A zigzag chain structure is formed, extending along (001). Each chain is surrounded by three others which are inter­connected through weak C=O⋯π_pyrid­yl_ [O⋯centroid = 2.999 (3) Å] and π_pyrid­yl_–π_pyrid­yl_ inter­actions [minimum ring centroid separation = 4.014 (2) Å], giving a three-dimensional framework.

## Related literature

For background to the coordination chemistry of pyridyl­ketone derivatives, see: Huang *et al.* (2003 ▶); Wan *et al.* (2008 ▶). For transition metal complexes of bis­(3-pyrid­yl)ketone, see: Chen *et al.* (2005 ▶, 2009 ▶); Chen & Mak (2005 ▶).
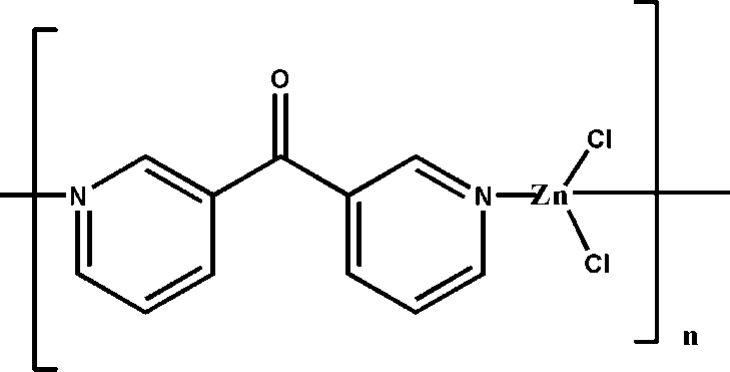


## Experimental

### 

#### Crystal data

[ZnCl_2_(C_11_H_8_N_2_O)]
*M* *_r_* = 320.46
Monoclinic, 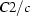

*a* = 9.9266 (7) Å
*b* = 15.5724 (10) Å
*c* = 7.8963 (6) Å
β = 93.878 (4)°
*V* = 1217.82 (15) Å^3^
*Z* = 4
Mo *K*α radiation
μ = 2.44 mm^−1^
*T* = 296 K
0.40 × 0.32 × 0.22 mm

#### Data collection

Bruker APEXII CCD area-detector diffractometer
Absorption correction: multi-scan (*SADABS*; Bruker, 2007 ▶) *T*_min_ = 0.913, *T*_max_ = 1.000
3481 measured reflections
1076 independent reflections
1041 reflections with *I* > 2σ(*I*)
*R*_int_ = 0.013

#### Refinement

*R*[*F* ^2^ > 2σ(*F* ^2^)] = 0.020
*wR*(*F* ^2^) = 0.055
*S* = 1.11
1076 reflections
79 parameters
H-atom parameters constrained
Δρ_max_ = 0.19 e Å^−3^
Δρ_min_ = −0.32 e Å^−3^

### 

Data collection: *APEX2* (Bruker, 2007 ▶); cell refinement: *APEX2* and *SAINT* (Bruker, 2007 ▶); data reduction: *SAINT*; program(s) used to solve structure: *SHELXS97* (Sheldrick, 2008 ▶); program(s) used to refine structure: *SHELXL97* (Sheldrick, 2008 ▶); molecular graphics: *SHELXTL* (Sheldrick, 2008 ▶); software used to prepare material for publication: *SHELXTL* and *PLATON* (Spek, 2009 ▶).

## Supplementary Material

Crystal structure: contains datablock(s) I, global. DOI: 10.1107/S160053681104671X/zs2159sup1.cif

Structure factors: contains datablock(s) I. DOI: 10.1107/S160053681104671X/zs2159Isup2.hkl

Additional supplementary materials:  crystallographic information; 3D view; checkCIF report

## Figures and Tables

**Fig. 1.**
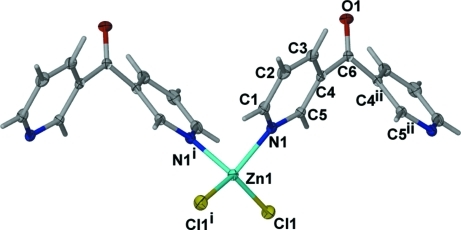
The title complex showing the atom-numbering scheme, with displacement ellipsoids shown at the 30% probability level. Hydrogen atoms are shown as spheres of arbitrary radius. Symmetry codes: (i) -x+1, -y, -z+1; (ii) -x+1, -y, -z.

**Fig. 2.**
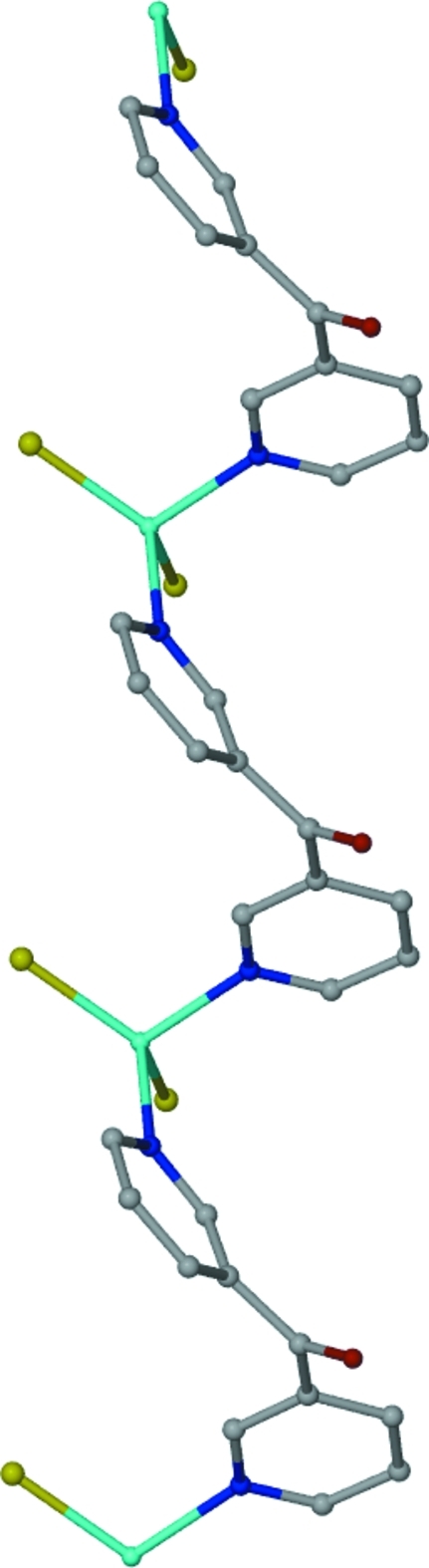
The helical chain structure of the title compound, extending along the c axial direction. All H atoms are omitted.

**Fig. 3.**
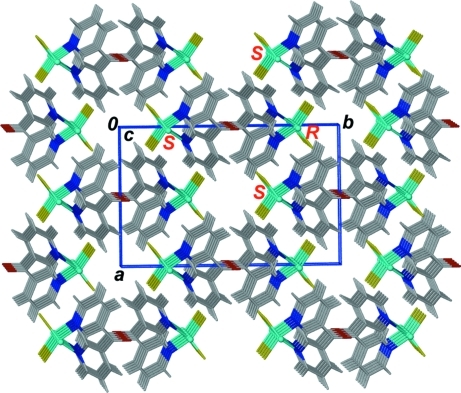
The packing structure of the title compound as viewed down the c axis of the unit cell.

## supplementary crystallographic information

### Comment

The carbonyl (C═O) group in pyridyl ketone derivatives produces versatile angular building blocks for use as ligands for the generation of various coordination supramolecular architectures (Huang *et al.*, 2003). With two pendant pyridyl rings and the rotatable C—C σ bonds, bis(3-pyridyl)methanone functions as an excellent µ_2_-bridging linker to assemble various transition metal salts into diverse coordination motifs, such as one-dimensional helical and zigzag chains (Chen & Mak, 2005), two-dimensional nets (Chen *et al.*, 2005), as well as honeycomb-like three-dimensional frameworks (Chen *et al.*, 2009).

Reported here is the structure of a new complex of bis(3-pyridyl)methanone with ZnCl_2_, the title compound [ZnCl_2_(C_5_NH_4_)_2_]_n_. In this complex, the  Zn^2+^ lies on a crystallographic twofold rotation axis and adopts a distorted tetrahedral stereochemistry [N1—Zn1—N1^i^ = 96.94 (8)°; Cl1—Zn1—Cl1^i^ = 122.25 (3)°: symmetry code (i) -*x*+1, -*y*, -*z*+1], with two chloride donors and two N donors from separate µ^2^-bridging bis(3-pyridyl)methanone ligands, in which the C═O group also lies on a twofold rotation axis (Fig. 1). This results in a zigzag chain structure extending along (001) (Fig. 2). Each helix is surrounded by three others which are interconnected through weak C6═O1···π_pyridyl_ interactions [O1···Cg1^iii^ 2.999 (3) Å] [symmetry code (iii) *x*+3/2, *y*+1/2, *z*+1) and weak π_pyridyl_···π_pyridyl_ interactions [ring centroid separation Cg1···Cg1^iv^ = 4.014 (2) Å] [symmetry code (iv) -*x*+3/2, *y*+1/2, -*z*+3/2] to form a three-dimensional framework (Fig. 3). For the  C═O···π_pyridyl_ contact, the O atom is embraced by two symmetry related pyridyl rings, similar to that found in [Cu(L)_2_(BF_4_)_2_] (Wan *et al.*, 2008) (C═O···centroid = 2.9–3.1 Å) [L = 2,6-pyridinediyl(bis(3-pyridinyl)methanone)].

### Experimental

The bis(3-pyridinyl)methanone ligand was obtained using the literature reaction procedure (Chen *et al.*, 2005). Reaction of this compound (19.1 mg, 0.1 mmol) with ZnCl_2_ (14.0 mg, 0.1 mmol) in methanol gave a colorless solution which after filtration, was allowed to stand in air for two weeks, gave colourless block-like crystals (yield 20.8 mg; 65%).

### Refinement

All H atoms were located in the difference electron density maps but were placed in idealized positions and allowed to ride on the carrier atoms, with C—H = 0.93 Å and with *U*_iso_(H) = 1.2*U*_eq_(C).

### Crystal data

**Table:**

[ZnCl_2_(C_11_H_8_N_2_O)]	*F*(000) = 640
*M**_r_* = 320.46	*D*_x_ = 1.748 Mg m^−^^3^
Monoclinic, *C*2/*c*	Mo *K*α radiation, λ = 0.71073 Å
Hall symbol: -C 2yc	Cell parameters from 254 reflections
*a* = 9.9266 (7) Å	θ = 2.6–25.0°
*b* = 15.5724 (10) Å	µ = 2.44 mm^−^^1^
*c* = 7.8963 (6) Å	*T* = 296 K
β = 93.878 (4)°	Block, colorless
*V* = 1217.82 (15) Å^3^	0.40 × 0.32 × 0.22 mm
*Z* = 4	

### Data collection

**Table:**

Bruker APEXII CCD area-detector diffractometer	1076 independent reflections
Radiation source: fine-focus sealed tube	1041 reflections with *I* > 2σ(*I*)
graphite	*R*_int_ = 0.013
ω scans	θ_max_ = 25.0°, θ_min_ = 2.6°
Absorption correction: multi-scan (*SADABS*; Bruker, 2007)	*h* = −11→11
*T*_min_ = 0.913, *T*_max_ = 1.000	*k* = −16→18
3481 measured reflections	*l* = −9→9

### Refinement

**Table:**

Refinement on *F*^2^	Primary atom site location: structure-invariant direct methods
Least-squares matrix: full	Secondary atom site location: difference Fourier map
*R*[*F*^2^ > 2σ(*F*^2^)] = 0.020	Hydrogen site location: inferred from neighbouring sites
*wR*(*F*^2^) = 0.055	H-atom parameters constrained
*S* = 1.11	*w* = 1/[σ^2^(*F*_o_^2^) + (0.0332*P*)^2^ + 0.7286*P*] *P* = (*F*_o_^2^ + 2*F*_c_^2^)/3
1076 reflections	(Δ/σ)_max_ < 0.001
79 parameters	Δρ_max_ = 0.19 e Å^−^^3^
0 restraints	Δρ_min_ = −0.32 e Å^−^^3^

### Special details

**Table:**

Geometry. All esds (except the esd in the dihedral angle between two l.s. planes) are estimated using the full covariance matrix. The cell esds are taken into account individually in the estimation of esds in distances, angles and torsion angles; correlations between esds in cell parameters are only used when they are defined by crystal symmetry. An approximate (isotropic) treatment of cell esds is used for estimating esds involving l.s. planes.
Refinement. Refinement of F^2^ against ALL reflections. The weighted R-factor wR and goodness of fit S are based on F^2^, conventional R-factors R are based on F, with F set to zero for negative F^2^. The threshold expression of F^2^ > 2sigma(F^2^) is used only for calculating R-factors(gt) etc. and is not relevant to the choice of reflections for refinement. R-factors based on F^2^ are statistically about twice as large as those based on F, and R- factors based on ALL data will be even larger.

### Fractional atomic coordinates and isotropic or equivalent isotropic displacement parameters (Å^2^)

**Table:**

	*x*	*y*	*z*	*U*_iso_*/*U*_eq_	
Zn1	0.5000	0.296457 (17)	0.7500	0.03157 (13)	
Cl1	0.35267 (5)	0.36506 (3)	0.57611 (6)	0.04896 (16)	
N1	0.59607 (15)	0.20836 (9)	0.60337 (18)	0.0307 (3)	
C2	0.77431 (19)	0.10656 (14)	0.5838 (3)	0.0444 (5)	
H2A	0.8584	0.0862	0.6246	0.053*	
C1	0.71626 (19)	0.17586 (13)	0.6597 (2)	0.0380 (4)	
H1A	0.7621	0.2011	0.7535	0.046*	
C3	0.7061 (2)	0.06820 (13)	0.4475 (2)	0.0411 (5)	
H3A	0.7422	0.0203	0.3966	0.049*	
C4	0.58196 (18)	0.10157 (11)	0.3857 (2)	0.0311 (4)	
C5	0.53146 (17)	0.17218 (11)	0.4663 (2)	0.0299 (4)	
H5A	0.4497	0.1955	0.4242	0.036*	
C6	0.5000	0.05441 (16)	0.2500	0.0340 (5)	
O1	0.5000	−0.02376 (12)	0.2500	0.0499 (5)	

### Atomic displacement parameters (Å^2^)

**Table:**

	*U*^11^	*U*^22^	*U*^33^	*U*^12^	*U*^13^	*U*^23^
Zn1	0.0417 (2)	0.02859 (19)	0.02354 (17)	0.000	−0.00413 (12)	0.000
Cl1	0.0606 (3)	0.0468 (3)	0.0376 (3)	0.0161 (2)	−0.0108 (2)	0.0043 (2)
N1	0.0368 (8)	0.0304 (8)	0.0245 (7)	−0.0003 (6)	−0.0006 (6)	0.0026 (5)
C2	0.0372 (10)	0.0514 (12)	0.0442 (11)	0.0107 (9)	0.0001 (8)	0.0125 (9)
C1	0.0397 (10)	0.0429 (10)	0.0307 (9)	−0.0023 (8)	−0.0033 (8)	0.0076 (8)
C3	0.0505 (11)	0.0366 (10)	0.0373 (10)	0.0142 (8)	0.0112 (8)	0.0071 (8)
C4	0.0428 (10)	0.0275 (9)	0.0236 (8)	0.0026 (7)	0.0057 (7)	0.0056 (6)
C5	0.0349 (9)	0.0288 (9)	0.0257 (8)	0.0025 (7)	0.0001 (7)	0.0037 (7)
C6	0.0494 (14)	0.0271 (13)	0.0268 (12)	0.000	0.0121 (10)	0.000
O1	0.0798 (15)	0.0254 (10)	0.0452 (11)	0.000	0.0094 (10)	0.000

### Geometric parameters (Å, °)

**Table:**

Zn1—N1^i^	2.0692 (15)	C1—H1A	0.9300
Zn1—N1	2.0692 (15)	C3—C4	1.395 (3)
Zn1—Cl1^i^	2.2123 (5)	C3—H3A	0.9300
Zn1—Cl1	2.2123 (5)	C4—C5	1.382 (2)
N1—C5	1.344 (2)	C4—C6	1.494 (2)
N1—C1	1.344 (2)	C5—H5A	0.9300
C2—C3	1.369 (3)	C6—O1	1.217 (3)
C2—C1	1.379 (3)	C6—C4^ii^	1.494 (2)
C2—H2A	0.9300		
			
N1^i^—Zn1—N1	96.94 (8)	C2—C1—H1A	118.7
N1^i^—Zn1—Cl1^i^	106.45 (4)	C2—C3—C4	119.40 (18)
N1—Zn1—Cl1^i^	110.92 (4)	C2—C3—H3A	120.3
N1^i^—Zn1—Cl1	110.92 (4)	C4—C3—H3A	120.3
N1—Zn1—Cl1	106.45 (4)	C5—C4—C3	118.33 (17)
Cl1^i^—Zn1—Cl1	122.25 (3)	C5—C4—C6	121.66 (15)
C5—N1—C1	118.25 (15)	C3—C4—C6	119.56 (16)
C5—N1—Zn1	121.04 (12)	N1—C5—C4	122.45 (16)
C1—N1—Zn1	119.81 (12)	N1—C5—H5A	118.8
C3—C2—C1	118.92 (17)	C4—C5—H5A	118.8
C3—C2—H2A	120.5	O1—C6—C4	119.45 (10)
C1—C2—H2A	120.5	O1—C6—C4^ii^	119.45 (10)
N1—C1—C2	122.60 (17)	C4—C6—C4^ii^	121.1 (2)
N1—C1—H1A	118.7		
			
N1^i^—Zn1—N1—C5	83.07 (13)	C2—C3—C4—C5	−0.7 (3)
Cl1^i^—Zn1—N1—C5	−166.32 (11)	C2—C3—C4—C6	−173.16 (16)
Cl1—Zn1—N1—C5	−31.20 (13)	C1—N1—C5—C4	2.2 (2)
N1^i^—Zn1—N1—C1	−85.85 (13)	Zn1—N1—C5—C4	−166.90 (12)
Cl1^i^—Zn1—N1—C1	24.76 (14)	C3—C4—C5—N1	−1.4 (2)
Cl1—Zn1—N1—C1	159.88 (12)	C6—C4—C5—N1	170.88 (15)
C5—N1—C1—C2	−0.9 (3)	C5—C4—C6—O1	−136.51 (12)
Zn1—N1—C1—C2	168.33 (14)	C3—C4—C6—O1	35.68 (17)
C3—C2—C1—N1	−1.2 (3)	C5—C4—C6—C4^ii^	43.49 (12)
C1—C2—C3—C4	1.9 (3)	C3—C4—C6—C4^ii^	−144.32 (17)

Symmetry codes: (i) −*x*+1, *y*, −*z*+3/2; (ii) −*x*+1, *y*, −*z*+1/2.
